# Challenges in Detecting Small Glass Particles: A Case Study of Persistent Forehead Swelling Post-surgery

**DOI:** 10.7759/cureus.88106

**Published:** 2025-07-16

**Authors:** Monica Potru, Jitesh Rawat, Kanika Khandelwal

**Affiliations:** 1 Department of Radiology, Dr. Rajendra Gode Medical College, Amravati, IND; 2 Department of Internal Medicine, Mayo Clinic, Rochester, USA

**Keywords:** foreign body detection, persistent swelling, post-traumatic swelling, radiological diagnosis, retained glass fragments

## Abstract

This case report presents a rare case of multiple retained foreign bodies following a trauma event, which were missed on initial surgical exploration and imaging. A 38-year-old man presented with a tender swelling on his right forehead that had been present for six months following trauma from a shattered glass photo frame which fell on his forehead. Initial imaging and local surgical exploration revealed a 2 cm glass fragment. Despite its removal, the patient continued to experience persistent tender swelling. A postoperative computed tomography (CT) scan identified additional hyperdense areas, which led to a second surgery and the discovery of multiple glass particles. This case highlights the challenges in detecting small glass fragments and emphasizes the importance of comprehensive imaging and clinical evaluation.

## Introduction

Laceration repairs following trauma often overlook retained foreign bodies, which can complicate wound healing and result in persistent symptoms. Imaging is conducted in only 11% of traumatic wounds, even though glass, a common foreign body, is radiopaque and detectable in 99% of cases if measuring 2 mm and 83% if measuring 1 mm [1]. Retained glass fragments can cause significant complications, including infection and delayed injuries. Effective assessment and management are essential to address these challenges. The possible reasons for a missed diagnosis in this case are the routine practice of visual inspections and conventional imaging in these cases, the uncommonly minute size of foreign bodies, and a lack of patient education. Clinicians should maintain a high level of suspicion for retained foreign bodies based on the type of injuries like these, caused by shattered objects. Clinicians should also consider the limitations of visual inspections and conventional imaging to detect foreign bodies. Educating the patient about the symptoms of retained foreign bodies and the possibility of retention will help them understand the need for a follow-up visit.

## Case presentation

**Figure 1:**
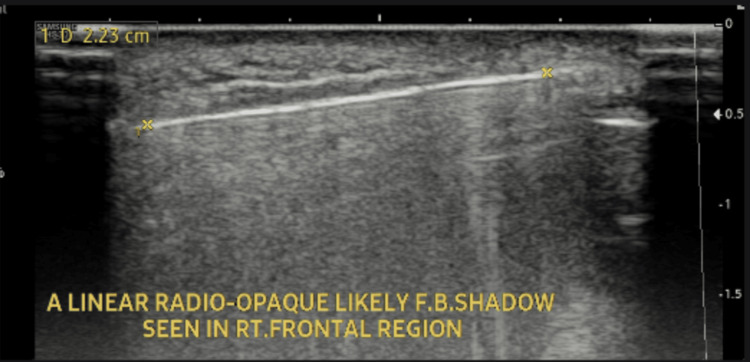
Ultrasound image with the thin linear hyperechoic area F.B.: foreign body; RT.: right

**Figure 2:**
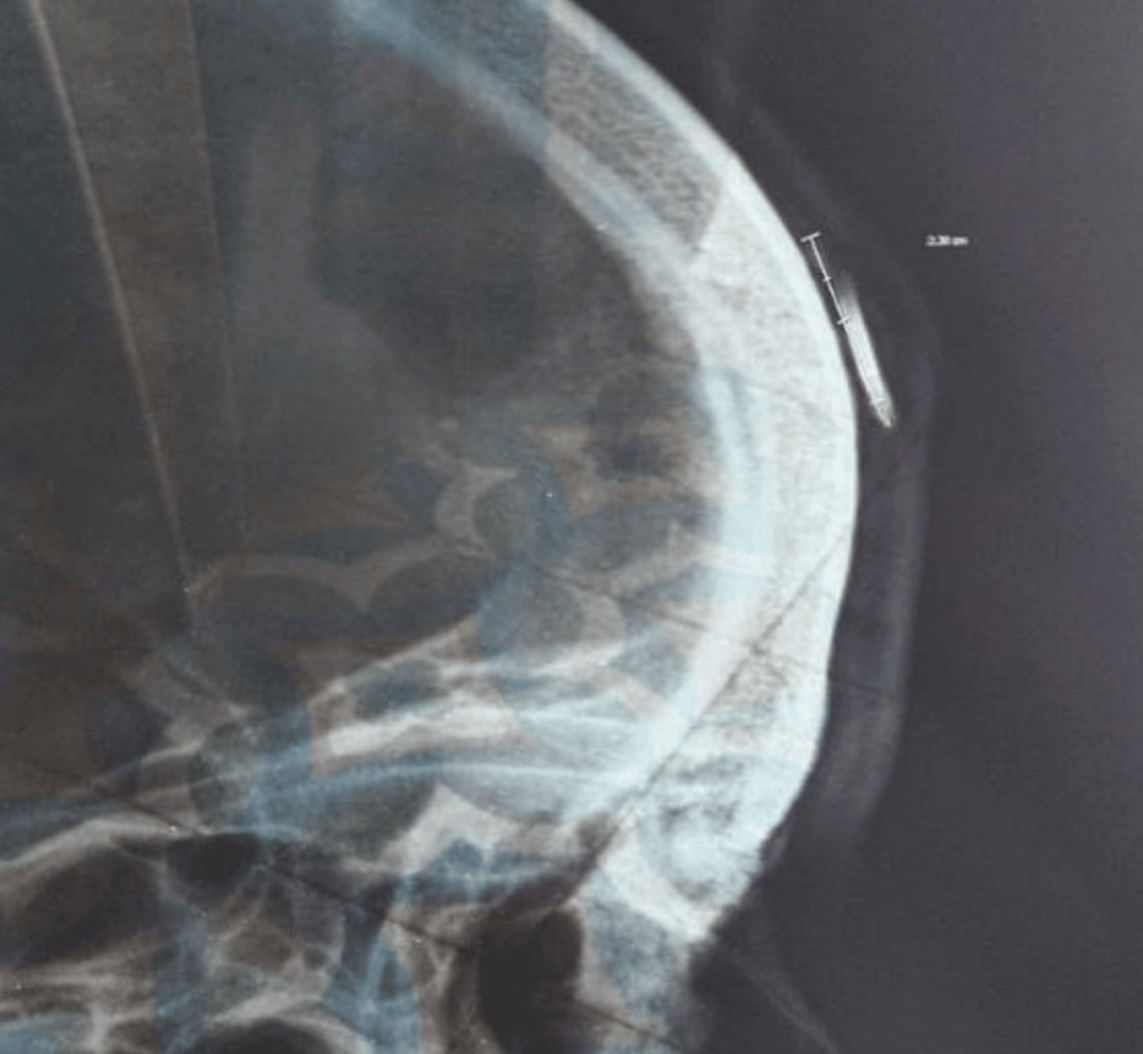
Lateral radiograph of the skull in the right frontal region (limited area radiograph)

**Figure 3:**
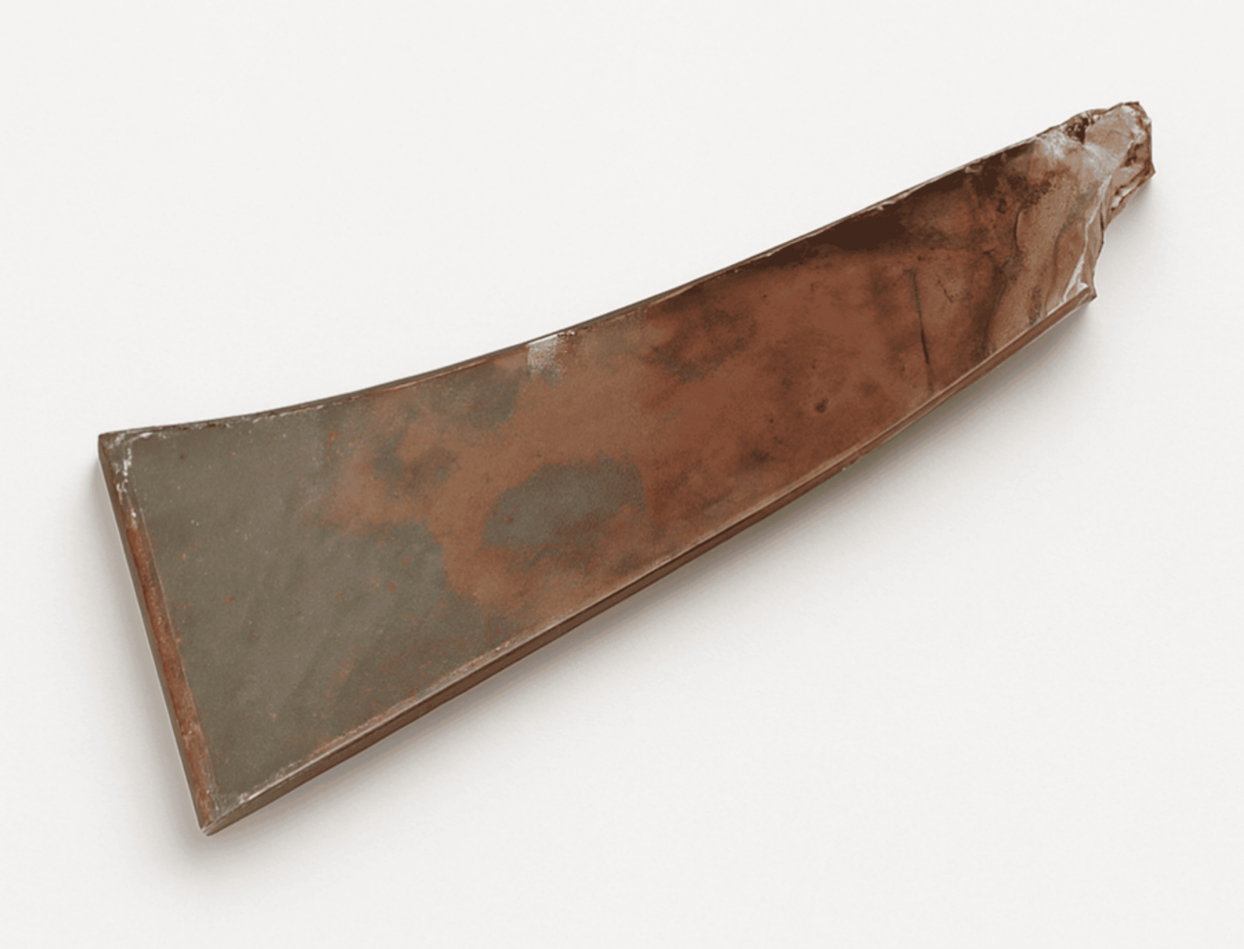
Glass particle in post-op image

A 38-year-old male patient with no significant past medical history, chronic diseases, or home medication use presented to the outpatient surgical department with persistent pain and swelling on his right forehead over the last six months. He had a history of an injury from a shattered photo frame that fell on his right forehead seven months ago. At the time of the incident, the patient was seen in the emergency department. The initial routine laboratory investigations had normal results. On physical examination, the patient had a laceration on the forehead, and a foreign body was suspected on examination, which was confirmed to be present in the subcutaneous tissue of the forehead by imaging. Ultrasound imaging identified a well-defined, thin, linear hyperechoic area in the subcutaneous tissue of the right frontal region, measuring 2.3 cm, with posterior acoustic shadowing and mild adjacent subcutaneous edema (Figure 1). X-ray imaging showed a linear dense radiopacity in the same region (Figure 2). Subsequently, the patient underwent surgical exploration under local anesthesia, a 2 cm glass fragment was then removed (Figure 3), and the laceration was repaired by suturing. A follow-up visit was scheduled after one week during this episode, but the patient missed it because he had no knowledge about its importance.

**Figure 4:**
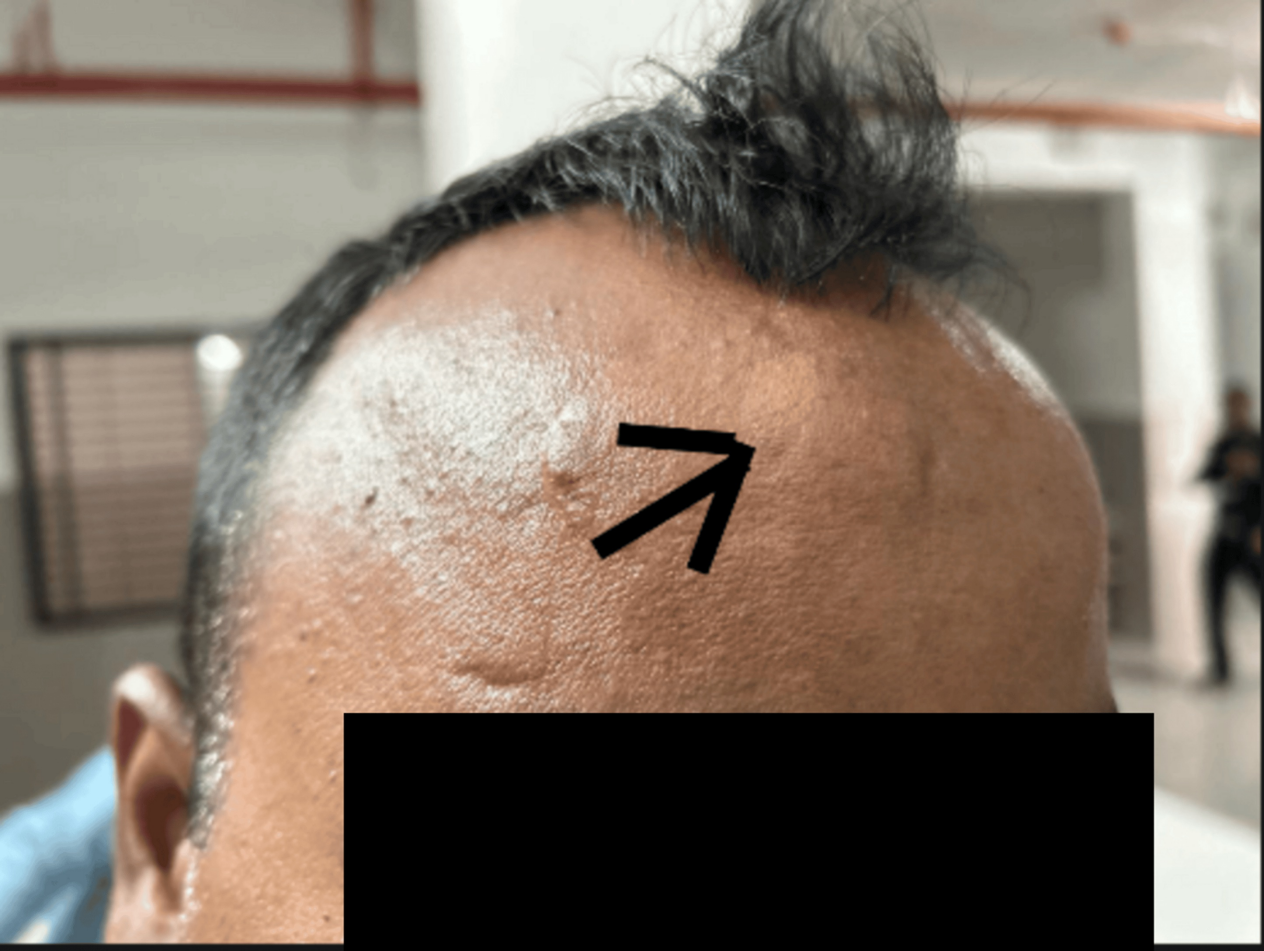
Right forehead swelling (arrow) with multiple scar marks

**Figure 5:**
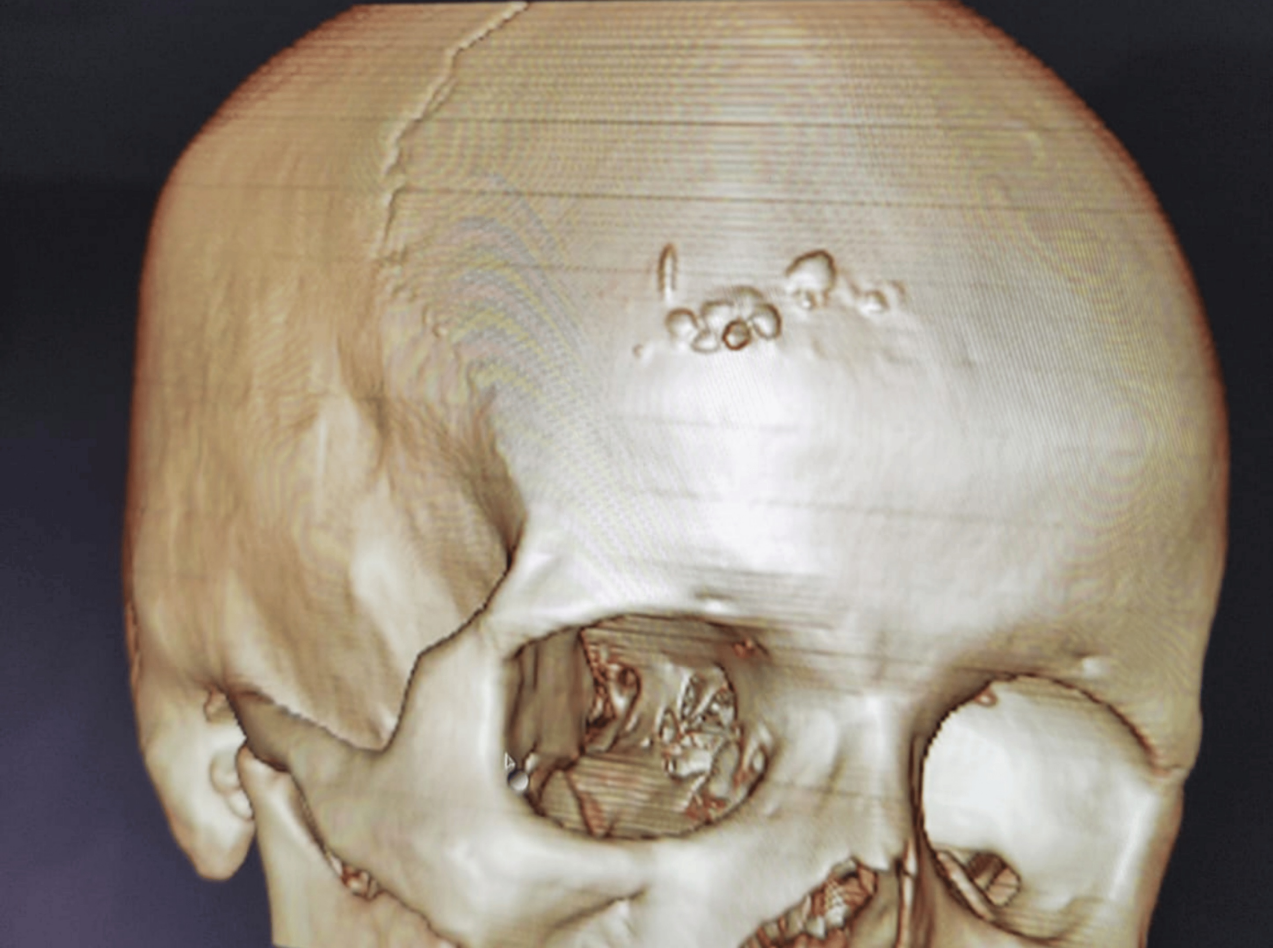
3D CT reconstruction image showing multiple irregularly hyperdense areas in the right frontal bone region CT: computed tomography

**Figure 6:**
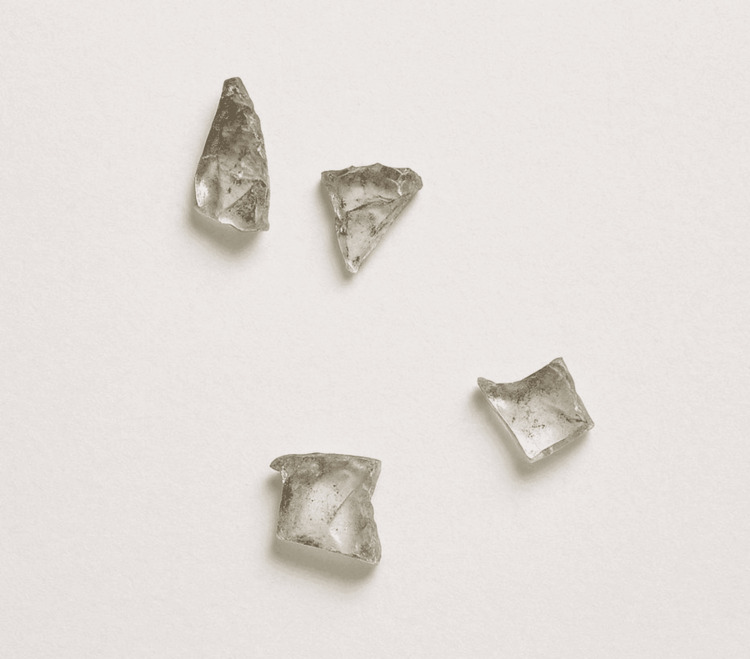
Post-op image showing multiple (i.e., four) broken glass particles

During the current visit, on physical exam, the swelling, measuring 2.5×1 cm (Figure 4), was tender, non-erythematous, and non-fluctuant. It has a normal overlying skin, and no discharge was noted. The patient had multiple facial scars from the previous trauma and a 1 cm sutured scar near the swelling. A computed tomography (CT) scan of the right forehead was done due to high clinical suspicion about retained foreign bodies, and it revealed multiple hyperdense areas in the subcutaneous tissue of the right frontal bone on 3D reconstruction (Figure 5). A second surgical exploration under location anesthesia, followed by suturing, was performed, uncovering additional glass particles (Figure 6). The patient is counseled about the importance of a follow-up visit, which was scheduled at the outpatient surgery unit after one week. The patient visited the outpatient unit; his swelling was noted to decrease in size, the pain resolved, and the sutured wound was healing well.

## Discussion

The patient had a foreign body sensation with retained glass, which is a symptom with underrated clinical significance. Sensation has a 31% positive predictive value and an 89% negative predictive value [2] for foreign bodies. Radiography detects only 57% of glass foreign bodies missed on visual inspection. Adequate exploration is crucial, potentially obviating the need for radiography; possible over-reliance on radiography can be a reason for this patient's condition. Further imaging in this patient would not only help in confirming the diagnosis and assessing foreign body characteristics but also guide management strategies [2]. For example, local anesthesia choice depends on wound location. The surgical technique varies depending on the shape and depth of the foreign body, emphasizing the importance of imaging [2]. Preparedness of both the clinicians and patients enhances removal success and minimizes complications. Setting removal deadlines is wise, taking into consideration the complications associated with retained foreign bodies; early consideration of CT or surgical referral would have provided better care for this patient. So, there is a need to do further studies on foreign body retentions and develop a proper management protocol. Patient education plays a significant role in these cases.

The type of foreign body also plays a significant role in diagnosis and management. Glass has a density of 2.4-2.8 g/cc, compared to water's density of 1 g/cc. This difference allows glass to be seen radiographically when embedded in soft tissues. Crystalware (containing 15-30% PbO) and some optical glass (containing 50-70% PbO) can have densities up to 5.9 g/cc due to their high lead content, resulting in images with greater contrast [3]. In routine clinical scenarios where the presence of glass in soft tissues needs to be confirmed, a well-positioned and properly exposed radiograph typically yields the diagnosis, and inserting a needle close to the foreign body can help in localizing the foreign body.

According to a study, ultrasonography successfully localized 19 out of 21 foreign bodies that could not be detected on radiographs. It can accurately determine the depth, size, and shape of the foreign body, as well as its proximity to anatomical structures like bones, tendons, blood vessels, or joints [2]. Foreign bodies will be seen as hyperechoic with varying degrees of shadowing and reverberation. Once the foreign body is located, its acoustic characteristics can provide information about its composition. For instance, gravel or wood will be seen as hyperechoic with pronounced posterior acoustic shadowing [4]. Acoustic reverberation causes metallic objects to look like a "comet tail" composed of distant, parallel lines. Variable acoustic shadows, like comet tails or diffuse beam scattering, can be caused by glass [4]. The imaging modality should be decided based on the type and size of the suspected foreign body (Table 1).

**Table 1:** Imaging techniques for detecting foreign bodies in the skin and subcutaneous tissue Table references: [2,4]

No.	Imaging modality	Objects that can be usually identified
1	Radiography	Bone fragments
		Certain fish spines
		Gravel and stones
		Metal fragments
		Pencil graphite
		Some types of plastic
		Teeth
		Wooden objects and thorns
		Glass
2	Ultrasonography	Metal fragments
		Pencil graphite
		Some types of plastic
		Stones
		Wooden objects
3	Computed tomography	Used for cases of unsuccessful exploration or infection

## Conclusions

The risk of infection increases in wounds with retained foreign bodies, particularly in older adults and patients with diabetes, leading to complications such as tetanus, granuloma formation, osteomyelitis, and delayed tendon or nerve injuries. Clinicians should maintain a high index of suspicion for foreign bodies, especially glass, in lacerations. A detailed examination and imaging are crucial for diagnosis, as glass fragments larger than 2 mm are usually detected by radiography, but smaller particles may require CT scans for accurate identification. Patient-reported sensations can also be significant indicators of retained foreign bodies. This case highlights the need for further studies related to foreign body injuries and the necessity to frame a management protocol for foreign body injuries. The limitations of radiographs and the advantages of going for appropriate imaging studies at the right time are discussed. The challenges faced in diagnosing foreign bodies also depend on the material they are composed of, and this should be taken into consideration for future diagnosis and treatment protocols. Patient education regarding the possibility of foreign body retention, its associated symptoms, and the need for follow-up is very important. In this case, the final diagnosis was a foreign body in the right forehead, with differential considerations including calcifications, scar tissue, fresh hematoma, and soft tissue emphysema.
